# Synthesis and applicability of reduced graphene oxide/porphyrin nanocomposite as photocatalyst for waste water treatment and medical applications

**DOI:** 10.1038/s41598-022-21360-8

**Published:** 2022-10-12

**Authors:** Ahmed M. El-Khawaga, Hesham Tantawy, Mohamed A. Elsayed, Ahmed I. A. Abd El-Mageed

**Affiliations:** 1 Department of Basic Medical Sciences, Faculty of Medicine, Galala University, Galala City, 43511 Suez Egypt; 2 Chemical Engineering Department, School of Chemical engineering, Military Technical College (MTC), Egyptian Armed Forces, Cairo, Egypt; 3 Chemistry Department, Faculty of Science, Galala University, Galala City, 43511 Suez Egypt; 4 Colloids and Advanced Materials Group, Chemistry Department, Faculty of Science, Minia University, Minia, 61519 Egypt

**Keywords:** Catalysis, Materials chemistry, Photochemistry

## Abstract

This study presents the synthesis and doping of reduced graphene oxide (rGO) with synthesized porphyrin (5,15-bisdodecyl porphyrin, C12P) nanoparticles to fabricate reduced graphene oxide-porphyrin (rGO-P) nanocomposite as well as demonstrates their outstanding removal activity of azo dye and antimicrobial potential. The synthesized porphyrin, rGO, and rGO-P nanocomposites were characterised using SEM, HRTEM, Raman spectroscopy, XRD, ^1^H-NMR, mass spectrometry, and UV–Visible spectroscopy. The ability of the synthesized rGO-P nanocomposite was then investigated (as catalyst and/or adsorbent) to impact its removal efficacy against Congo red (CR) as a well-known toxic, mutagenic and carcinogenic synthetic dye. The findings indicated that 0.01 g of rGO-P nanocomposite achieved 78.0% removal of CR at pH 3.0. Besides, the removal efficacy was evaluated while studying many aspects i.e. pH, CR initial concentration, and rGO-P nanocomposite amount. Moreover, the minimum inhibitory concentration (MIC) and zone of inhibition (ZOI) of antimicrobial activity against pathogenic bacteria and yeast were evaluated. The antimicrobial results showed that rGO-P nanocomposite revealed the greatest antimicrobial activity against *Candida albicans, Enterococcus faecalis,* and *Staphylococcus aureus* with ZOI values of 24.3, 21.8, and 22.1 mm, respectively. Consequently, it demonstrates the substantial potential of rGO-P nanocomposite in the effective removal of pollutant dyes as well as significant antibacterial and antifungal properties.

## Introduction

Because of their remarkable physical and chemical features, porphyrins and other similar compounds have recently gained a lot of attention and have been widely explored. They are not only significant as natural photocatalysts but also as major contenders in several other domains (i.e., electronic devices, physics, and many materials science applications)^1–9^ due to their simplicity of synthesis, which includes a wide range of substituents.

The chemical reduction treatment may remove numerous functional groups from graphene oxide, resulting in graphene sheets with reduced functional groups, also referred to as reduced graphene oxide (rGO)^10,11^. Despite having poorer electrical conductivity than graphene sheets, rGO is regarded as a flexible substance for photocatalysis. Furthermore, because of its π–π interactions, lower oxygen content, larger surface area, higher hydrophobicity, and more defect sites, rGO can easily form stable aqueous dispersion and has a higher adsorption capability for aromatic pollutants^12^.

Polluted water from many industries i.e. textile, leather, pigments, rubber and plastic contains residual synthetic dyes^13^. Several types of the used synthetic dyes are toxic and nondegradable pollutants. Their presences in natural water caused several environmental and health problems^14^. One of the common used synthetic dyes in textiles, rubber, plastics, and cosmetics is Congo red dye^15^. Congo red is considered toxic, mutagenic and carcinogenic synthetic dye. It is characterized by a stable chemical structure which reduces the efficiency of several traditional decolorizing methods upon the releasing of it into the aquatic environment^16^. This, in turn, affects negatively on the aquatic life and even the food chain. Several technologies have been evaluated to eliminate synthetic dyes in water as membrane filtration, flocculation, ion exchange, electrochemical destruction, electrokinetic coagulation, ozonation, adsorption, biodegradation, photocatalytic degradation and Fenton's oxidation^17,18^.

Photocatalysis is regarded as one of the primary approaches for adopting green chemistry processes owing to its reduced environmental risks, process safety, and low energy demands^19^.

The integration of graphene into the composite may result in the emergence of new design and development opportunities for next-generation catalysts. Therefore, the functionalization of rGO might open the way for its use in a variety of practical purposes. Since the presence of nanoparticles anchoring at the graphene surface enhances its photocatalytic efficacy for effective pollutant degradation under visible light irradiation^20^. Therefore, anchoring graphene with porphyrin via covalent or non-covalent bonds will result in efficient heterogeneous interfacial electron transfer and long-distance transport at the surface junction; thus, it is anticipated that the catalytic activity of the resulting nanocomposite will be tuned.

Nanocomposites of graphene oxide and porphyrin (GO-P) are noteworthy because they combine the outstanding features of two distinct materials. The composite performance was enhanced by combining graphene's unique characteristics and the porphyrin on its surface. In particular, using graphene as porphyrin support led to a quantifiable improvement in the electrical characteristics and synergistic interactions of porphyrin and graphene. Literature has several examples of graphene oxide that has been functionalized with porphyrins and their metal complexes^21–23^.

Accordingly, it was proposed that the integration of porphyrins with rGO could lead to multifunctional carriers having promising characteristics and versatile functional components. From this perspective, porphyrin-based rGO are ideal candidates and their full potential has not yet been tapped.

Many reported studies are focused on the integration between carbonic materials (i.e. graphene oxide, carbon nanotubes) and other nanoparticles (i.e. magnetite, TiO_2_ and CeO) as a powerful adsorbent used in the removal of different pollutants i.e. (Acid Brown‑14, Indigotin blue dye and Antimony III and V)^24–26^. Recently, the antibacterial properties of various materials, including metal ions^27,28^, carbon nanotubes (CNTs)^29,30^ and graphene-related materials^31^, have also hold great promises in health and environmental applications.

In this study, we report an approach for the non-covalent functionalization of free-base porphyrin (5, 15-bisdodecylporphyrin, C12P) with reduced graphene oxide surface to produce nanocomposite of reduced graphene oxide-porphyrin (rGO-P). Multiple spectroscopic methods accurately described the resultant nanocomposite. The spectrophotometric evaluation of the catalytic activity of the resultant porphyrin-graphene oxide nanocomposite for the catalytic reduction of Congo red (CR) dye was also conducted. In addition, we discussed the potential mechanism behind the reduction process. Finally, we discussed the antimicrobial activity of the synthesized rGO-P nanocomposite against some pathogenic bacteria and fungi.

## Experimental section

### Chemicals

Graphite 99.5% (NICE / India), Potassium Permanganate 98% (Alpha Chemicals / India), Ascorbic Acid 98% (Alpha Chemicals / India) and Congo red ≤ 100% (Aldrich/Germany) were used. All the chemicals used in the experimental work are in the reagent grade. They were obtained from the indicated sources, with some specified purities, with no further purification, as shown in Table S1. The porphyrin purification was performed using column chromatography (with silica-gel, i.e., 63–200 µm, neutral, spherical, Kishida Chemicals Co., Ltd.) as well as a recrystallization process.

### Synthesis of porphyrin (5, 15-bisdodecylporphyrin, C12P) nanoparticles

We synthesized the target porphyrin molecule using the procedures reported in our previous study^32^, in which 1 g of dipyrromethene (5 mmol) was dissolved in anhydrous dichloromethane (1.1 L) where the flask was kept in dark conditions under nitrogen atmosphere for 20 min. Afterward, a solution of tridecanal (0.7 g, 4.7 mmol dissolved in 20 ml CH_2_Cl_2_) was dropwisely added to the dipyrromethene solution. After 10 min, 83 µl of trifluoroacetic acid was added dropwise into the reaction mixture. Under the ambient conditions, the reaction mixture was kept under stirring for 24 h. After that, we added *p*-chloranil (1.7 g, 7 mmol) with an extra stirring for 2 h, followed by neutralizing the solution by adding 3 ml of triethylamine (Et_3_N). Flash column chromatography was then used to purify the product (Hexane/CH_2_Cl_2_: silica gel = 1:1), followed by recrystallization with CH_2_Cl_2_/excess MeOH, giving the final target molecule in the form of red–purple powder (with a yield of 0.3512 g, 23%). All the synthesis procedures were obviously depicted in Fig. S1.

### Reduced graphene oxide (r-GO) nanosheets' synthesis

Chemical oxidation and exfoliation of the graphite precursor were carried out via an improved hummer method in a similar approach as previously reported^33–35^. Typically, in a 600 ml concentrated sulfuric acid (H_2_SO_4_) and 75 ml of concentrated phosphoric acid (H_3_PO_4_), 5 g of fine graphite powder was added to a 2 L beaker kept at room temperature. Afterward, 30 g of potassium permanganate (KMnO_4_) was gradually added to the mixture stepwise with continuous stirring for 1 h. Further, the final obtained mixture was kept under stirring for twenty-four hours at ambient temperature. Later, we added the obtained dense mixture in portions to 4 L of cold distilled water in a 5 L beaker, which was kept at 2 °C in the water path under continuous stirring. Subsequently, the yellowish color of the successful graphene oxide (GO) product was observed as 100 ml of 15% hydrogen peroxide was added to the ending diluted mixture. The product was left for decantation and rinsed three times with 1 M HCl acidified water, followed by three times with distilled water to neutralize.

Regarding the reduction step, the obtained washed GO was transferred to a 2 L beaker, and the reduction process was carried out by employing 10 g of ascorbic acid as a reducing agent. It was introduced to the GO dispersion in steps while vigorously stirring at 80 °C, and the solution was left for 24 h. Finally, the obtained black reduced graphene oxide (rGO) product was washed with distilled water three times before vacuum filtration. The obtained rGO was separated and washed via vacuum filtration with distilled water followed by ethanol to remove any remnant byproducts or impurities. Finally, the obtained powder of rGO was desiccated for 24 h at ambient temperature, then put for another 24 h in an oven heated to 40 °C. All the synthesis procedures were obviously depicted in Figure S2.

### Preparation of reduced graphene oxide-porphyrin (rGO-P) nanocomposite

We prepared the rGO-P nanocomposite as depicted in Figure S3. Firstly, 0.33 mg of porphyrin was dissolved in CHCl_3_ (20 ml). After sonicating for 10 min, 3 mg of rGO was added to the solution with continuous sonication for 5 h. The resultant suspension was kept for 2 h to be sedimented. After removing the top 5% of the supernatant by decantation, filtration was performed using a membrane filter of 0.1 µm mesh (MILLIPORE). To remove the non-reacted porphyrin, the precipitate was rinsed with 100 ml of CHCl_3_. Finally, the resultant composite was dried under vacuum and kept for further usage.

### Characterization techniques

SEM technique was utilized to investigate the morphology of initial graphite particles (EVO-MA10, ZEISS), while TEM measurements were done using JEM-2100F, JEOL, Japan. XRD was used to examine the crystal structure (XRD, D8 Advance, Bruker Corporation, Germany). Using a dispersive Raman microscope, rGO powder samples were subjected to Raman spectroscopy (model Senterra II, Bruker, Germany). The continuous collection of Raman spectra with a 4 cm^− 1^ of a spectral resolution was analyzed spectroscopically. The Raman excitation source was focused using a Nikon 20 objective (10 mW, 532 nm neodymium-doped yttrium aluminum garnet (Nd: YAG) laser- Bruker, Germany). The data collecting time was kept at 1000 ms (co addition 3) for the 50 × 50 μm diaphragm illumination zone.

Furthermore, a third-order polynomial was used to correct the fluorescence baseline, which was then followed by the use of a three-point moving average filter to remove the majority of the perturbing baseline and increase the signal-to-noise ratio. Using the Jasco V530 spectrometer, the UV–Vis absorption spectra of rGO were determined (Japan). A UV–Vis spectrophotometer (Agilent Technologies Cary 60 UV–Vis) was used to measure UV–Vis spectroscopy. The instrument response and background were used to standardize the acquired UV–Vis absorption spectra. ^1^H NMR was performed using an NMR spectrometer (500 MHz, JEOL, Japan). As an internal standard, tetramethylsilane (TMS) was used to adjust the NMR signals. Using a Shimadzu AXIMA-CFR MALDI-TOF mass spectrometer, mass spectrometric measurements were performed.

### Photocatalytic degradation of Congo red (CR) using rGO-P nanocomposite

About 10 mg of the rGO-P nanocomposite was dispersed in 50 ml of an aqueous CR solution (with a beginning concentration of 10 mg/l in a separate 125 ml beaker) with continuous stirring under ambient temperature (25 °C) and in the dark for 90 min. The procedure was repeated until equilibrium between adsorption and desorption was reached between CR and the produced photocatalysts. The photocatalytic activity of these nanomaterials was evaluated separately under ultraviolet irradiation for degrading CR dye. A syringe fitted with a filter (2.5 mm pore size) was utilized for pulling out a specimen of the CR suspension at consistent time intervals of irradiation (1 ml). Utilizing a UV–Vis spectrophotometer at λ_max_ = 490 nm, the degradation rate of CR was estimated by measuring the change in CR concentration vs. irradiation time. As a reference, DI water was utilized.

### Antimicrobial properties

Using the agar-disc distribution method, the antibacterial performance of the produced samples (20.0 μ g/ml) was evaluated^36^, towards a gram-negative bacteria, i.e. [*Escherichia coli* (ATCC 25922) and *Shigella sonnei* (ATCC 29531)] and a gram-positive bacteria, i.e. [*Staphylococcus aureus* (ATCC 25923) and *Enterococcus faecalis* (ATCC 29212)] in addition to fungi, i.e. *Candida albicans* (ATCC 14053). Conventional antibiotic discs (E; 20 μg/ml; 6.0 mm diameter) were selected to assess the effectiveness of the synthesized samples.

The technique of Luria–Bertani (LB) medium's serial dilutions was used to measure the investigated substances' minimum inhibitory concentrations (MIC) with the greatest antibacterial activity^36^. For these measurements, a negative control consisting of the medium broth and a positive control consisting of the studied pathogenic microorganisms, the utilized medium broth, and synthetic samples (starting at a concentration of 20.0 μg/ml) were employed. After incubation at 36.0 ± 1.0 °C for 24 h, the MIC was measured^37^. SPSS version 15 software was employed to calculate ONE WAY ANOVA, Duncan's multiple series, and the least significant difference (LSD) for statistical analysis of the results^38^.

## Results and discussion

### The porphyrin molecule's synthesis and characterization

**Figure 1:**
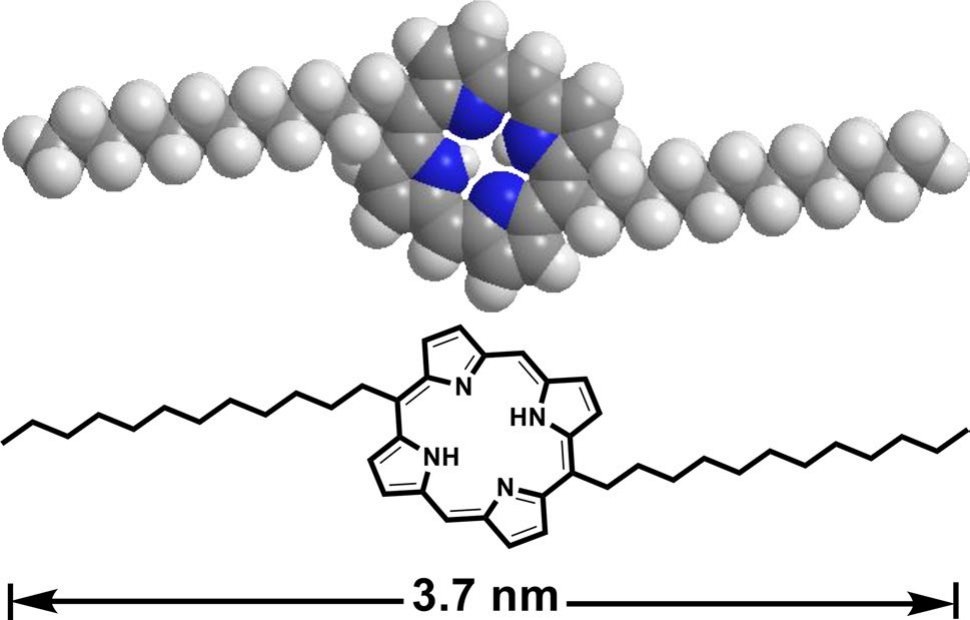
The chemical structure of 5, 15-bisdodecylporphyrin (C12P).

The target porphyrin molecule (Fig. 1) was synthesized as shown in Figure S1. The synthesis was successfully confirmed using different characterization techniques, i.e. ^1^H NMR, UV–Vis, as well as high-resolution mass spectrometry. The ^1^H NMR spectrum (Figure S4) shows the characteristic NH singlet peak (δ = − 2.92 ppm), *meso*-H singlet peak (δ = 10.16 ppm), and also *β*-pyrrole-H doublet peaks (δ = 9.57 and 9.41 ppm). Figure 3 presents the characteristic Soret peak at λ_max_ = 410 nm, in addition to four Q-bands at 504, 535, 577, and 632 nm. The molecular mass of the C12P molecule (C_44_H_62_N_4_) found for [M^+^] is 646.50; however, the high-resolution molecular mass found for [M + H]^+^ is 647.5048, as clearly shown in Figures S5 and S6, respectively.

### Characterization of the synthesized rGO

**Figure 2:**
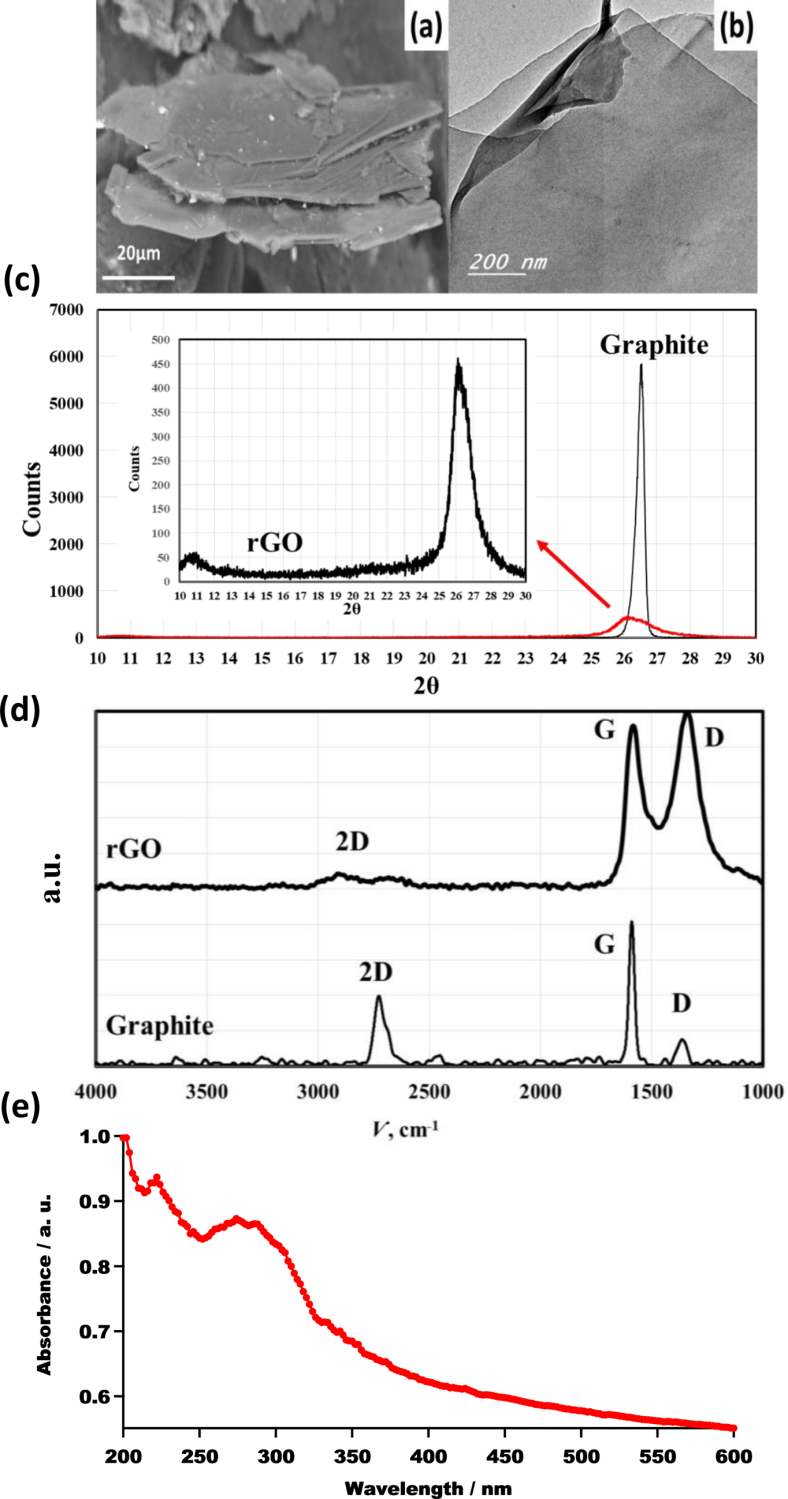
(**a**) SEM micrograph of the graphite precursor, (**b**) TEM micrograph of the produced rGO. (**c**) XRD diffraction pattern of the synthesized rGO. (**d**) Raman spectra of produced rGO compared to graphite precursor. (**e**) UV–Visible absorption spectrum of synthesized rGO.

Scanning electron microscopy (SEM) was used to characterize the graphite precursor. As seen in Fig. 2a, the noticed morphological profile of the used graphite corroborated its flake nature. Furthermore, the SEM micrograph revealed a 150 µm-long structure with numerous layers.

Moreover, the effective exfoliation of graphite to rGO was shown by TEM micrograph of synthesized rGO (Fig. 2b). Furthermore, on the surface of rGO, there are evidently no leftover reactants or byproducts.

The obtained diffraction patterns of the incident beam demonstrated the disappearance of the prominent peak at 2θ ~ 26.5° of graphite precursor in the rGO sample, as represented in Fig. 2c. The vanishing of the plane (002) characteristic for the well-ordered graphite hexagonal structure within rGO confirms the complete successful conversion of graphite to rGO without any detectable graphite impurities^39,40^.

Figure 2d depicts the obtained Raman spectrum of produced rGO compared to graphite at the beginning. Raman spectra of rGO revealed a G band at 1582 cm^− 1^ (typical of graphite) and a wide D band at 1350 cm^− 1^ (typical of rGO); this distinctive peak validated the production of rGO^41–45^.

As shown in the TEM, Raman, and XRD data, rGO has lost its initial graphite structure^43^. GO reduction produces sheets of rGO that are clustered and randomly packed. The development of (002) with a wide and low-intensity XRD peak centered at 2θ ≈ 26.5° (d spacing of 3.380 Å ) confirmed the sequential production of extremely thin rGO layers as a result of a high degree of exfoliation^39,40^. The data obtained from Raman spectra approve the successive synthesis of reduced graphene oxide with few-layer measuring < 10 nm in thicknesses^41,44,45^.

Figure 2e illustrates the UV–Visible absorption spectrum of synthesized rGO. Absorption of rGO at 284 nm is attributable to π → π * transitions of aromatic C–C bonds^45,46^. This suggested the creation of structures with a high level of conjugation^47^. The low peak detected at 224 nm is attributable to n → π* transitions caused by any remaining oxygen-containing functional groups^48^.

### Preparation and characterization of rGO-P nanocomposite

**Figure 3:**
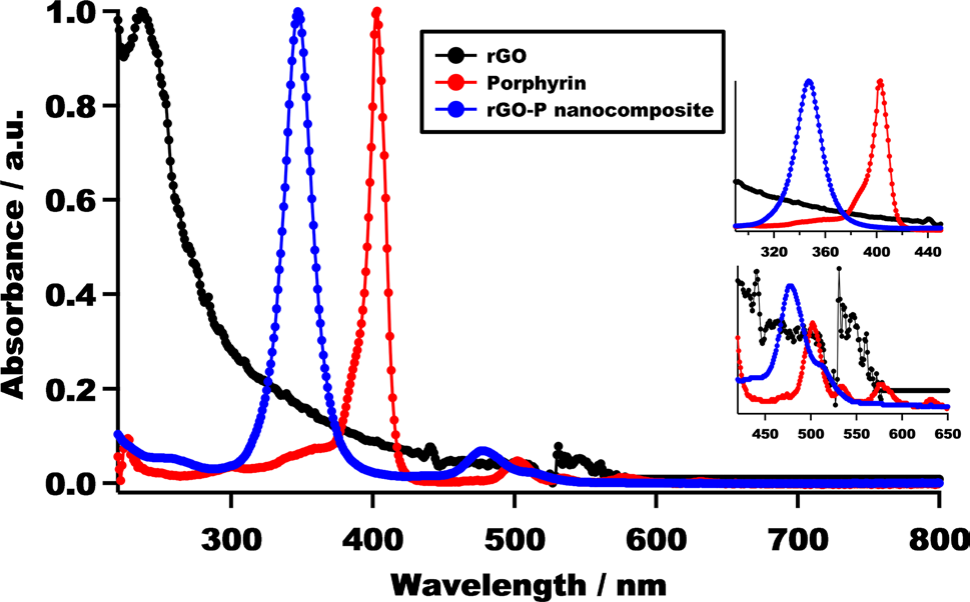
UV–Visible spectrum (measured in CHCl_3_) of raw rGO, porphyrin, and rGO-P nanocomposite. The inset shows the blue shift of the Soret peak as well as Q-bands after the nanocomposite formation.

The rGO-P nanocomposite was fabricated by mixing porphyrin solution with rGO under sonication. The resultant composite was characterized using UV–Vis spectroscopic measurements to confirm the interaction between porphyrin and rGO. Consequently, we studied the UV–Vis spectra of rGO and porphyrin molecules before and after complexation. Figure 3 represents the UV–Vis spectra of the porphyrin molecule, rGO, as well as rGO-P, in which there is an obvious blue shift in the composite spectrum compared to the porphyrin spectrum. The Soret peak was blue-shifted (402 → 347 nm) in addition to the Q-bands (502 → 477 nm), as depicted in Fig. 3. This blue shift is possibly due to the non-covalent interaction (i.e., π–π stacking) between C12P and rGO, which agrees with the reported results^32,49–52^.

**Figure 4:**
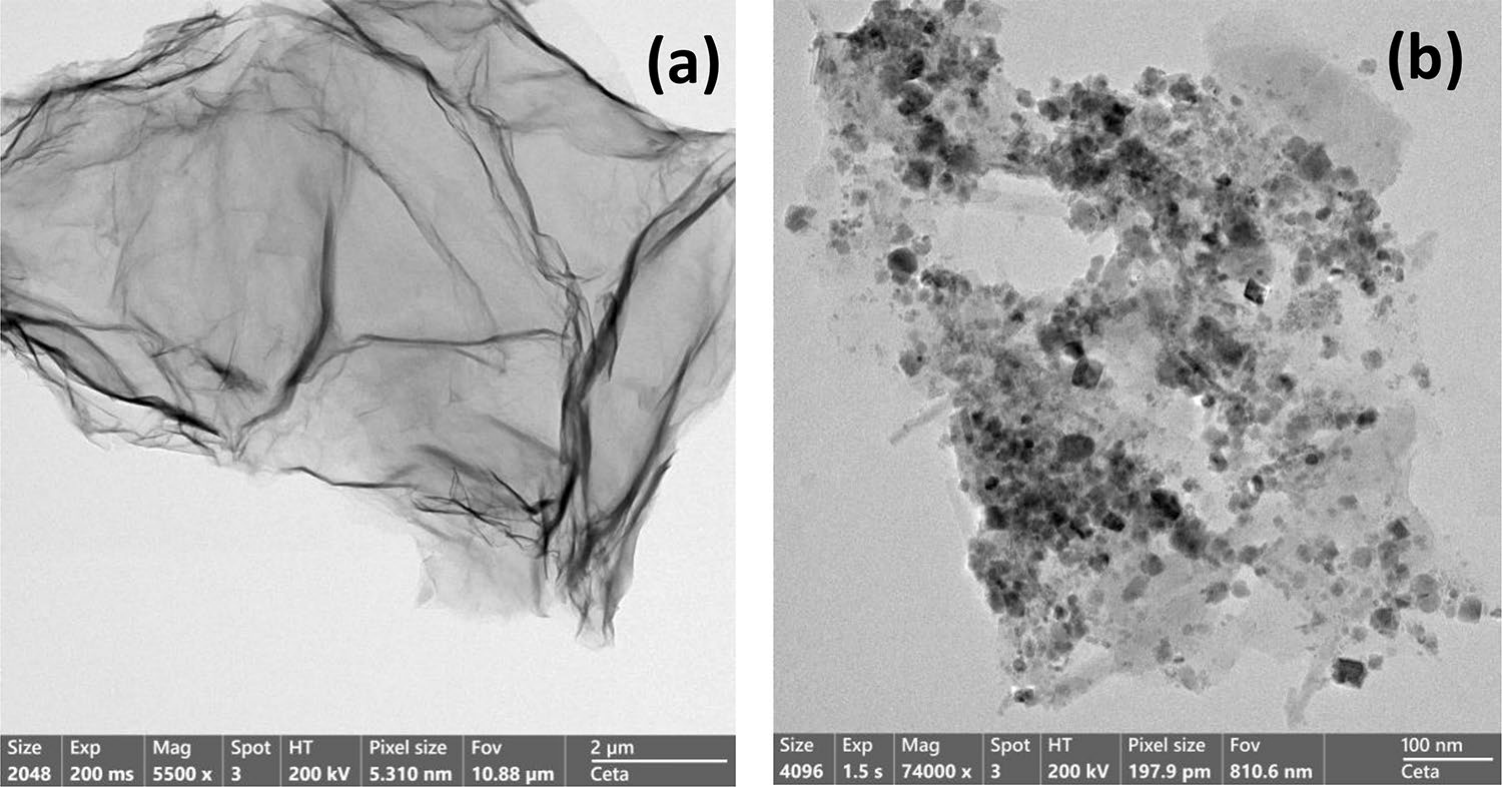
HR-TEM micrographs of (**a**) rGO and **(b**) rGO-P nanocomposite.

The rGO-P nanocomposite was also characterized using high-resolution transmission electron microscopy (HRTEM). Figure 4 shows the HR-TEM images rGO before and after the complexation with porphyrin. The black dots in Fig. 4b represent the loaded C12P molecules over the rGO surface, confirming the π–π stacking interactions between C12P molecules and rGO sheets.

### Removal performance of rGO and rGO-P nanocomposite toward Congo red (CR) dye

**Figure 5:**
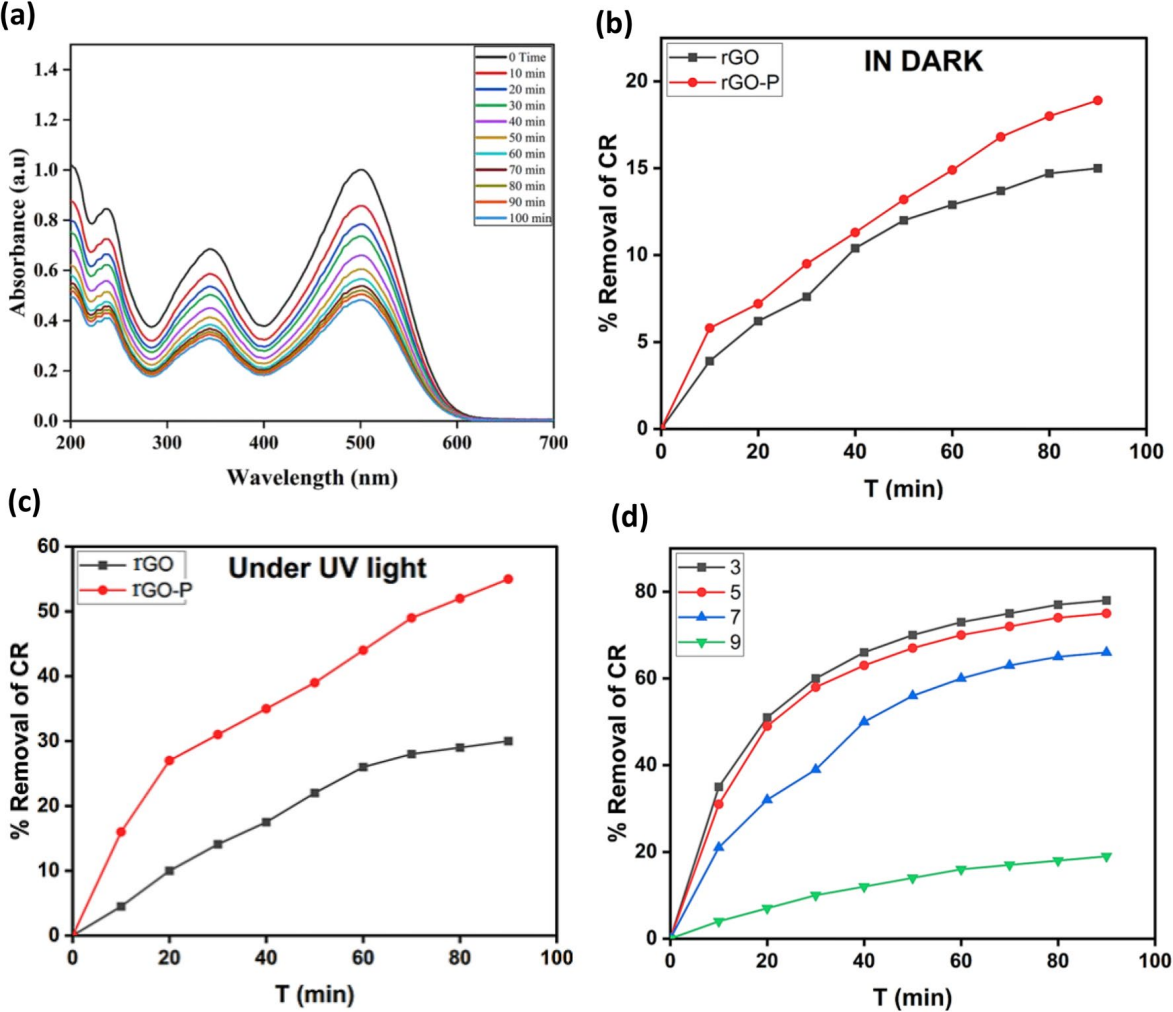
(**a**) UV–Visible spectra of Congo red (CR) at different concentrations with time intervals. (**b**) Removal percentage of CR within 90 min due to adsorption activity of rGO and rGO-P composite. (**c**) Removal percentage of CR by rGO and rGO-P nanocomposite as a function of time under UV irradiation. (**d**) Variation of CR removal (%) with time at different solution pH i.e. 3.0, 5.0, 7.0 and 9.0.

The elimination of CR has been evaluated spectrophotometrically at the dye's highest absorption (viz. λ_max_ = 490 nm, Fig. 5a), which matched well with early-declared findings^53^.

CR is a potentially carcinogenic substance and may cause irritation, dermatitis, and conjunctivitis. Moreover, its consumption might result in gastrointestinal discomfort, headache, diarrhea, and vomiting^54^. In order to examine the photocatalytic activity of the synthesized photocatalysts, CR was used as a model contaminant in this work. Figure 5b shows the adsorption efficiency of CR by using virgin rGO and rGO-P nanocomposite under dark conditions. The bare rGO shows only 15% after 90 min adsorption of CR, while the adsorption efficiency of CR without any light source after adding rGO-P was 18.9% after the same time.

The as-synthesized rGO-P nanocomposite surface has plenty of aromatic rings which are rich in π-electrons. Thus, it is an ideal surface for extraction of benzene-based dye (CR) by π–π interactions. Moreover, rGO-P nanocomposite surface consists of various –OH groups, while the CR molecule has various electronegative atoms like O, N, and S. These –OH groups can actively participate in hydrogen bonding, ergo efficiently bind with the CR molecules and facilitate its removal by adsorption in the dark^55^.

#### Synergistic catalytic effect of rGO-P nanocomposite under UV

After the adsorption, the CR discharge system incorporating rGO was exposed to UV light, and rGO-P was added as a photocatalyst. According to Fig. 5c, the addition of rGO and rGO-P to the CR solution under UV light irradiation boosted the CR removal effectiveness by up to 30 and 57 percent after 90 min, respectively. Comparing the findings of light absorption under dark and light irradiation conditions revealed that photocatalytic degradation by the rGO and rGO-P nanocomposite was responsible for the majority of the CR elimination effects. The removal performance due to the high photon energy in UV light and the impact of the designed metal–semiconductor heterojunction of the nanocomposite, which allows charge separation and absorption of incoming light, the nanocomposite's efficiency under UV reached up to 57 percent after 90 min. The photocatalytic activity by rGO and rGO-P nanocomposite is explained below in the mechanism section.

#### Effect of pH on removal of CR

One of the most critical elements for removal studies is the pH of the solution. The influence of starting pH values of CR solution was investigated for 90 min under defined experimental settings (10 mg of the produced nanocomposite, 50 ml of a 10 mg/L CR solution, and T = 25 °C). Figure 5d is a graph depicting the fluctuation of CR elimination (percent) with time at varying solution pH (3.0–9.0). The highest equilibrium CR removal was recorded at pH 3.0 i.e. 78%.

To determine the point of zero charges (PZC) of the rGO-P nanocomposite, 0.01 g was added to a 50 mL solution of 0.01 M NaCl. HCl or NaOH was used to adjust the pH levels of the solutions to 2, 4, 6, 8, 10, and 12. For 48 h, the samples were agitated at 200 rpm. After separating the rGO-P nanocomposite, the pH values of the solutions were measured. Using a graph that compares the end pH to the beginning pH, the pH of the PZC value was calculated. The pH of the PZC was found to be 6.9 (where there is no significant change between final and starting pH readings) as depicted in Fig. S7. This indicates that the rGO-P surface is positively charged when pH < pH_PZC_ and negatively charged when pH > pH_PZC_. In addition, when the pH_(solution)_ = the pH_PZC_, the surface charge of the photocatalyst is neutral, and there is a negligible electrostatic attraction between the ions (CR ions) and the surface of the photocatalyst^56^. The pH_PZC_ with respect to rGO-P was found to be 6.9, and this finding explained why the photocatalytic degradation of CR was greatest at pH 3.0, as shown in Fig. 5d. Therefore, at this stage, the net surface charge of the rGO-P nanocomposite is positive, attracting the negative charge of CR and enhancing the photocatalytic destruction of CR. At pH > 7.0, the photocatalytic degradation of CR started to decrease owing to the repulsion forces between the negative charge of CR and the negative net surface charge of the rGO-P nanocomposite at pH > 6.9.

#### Initial concentration's effect of CR

**Figure 6:**
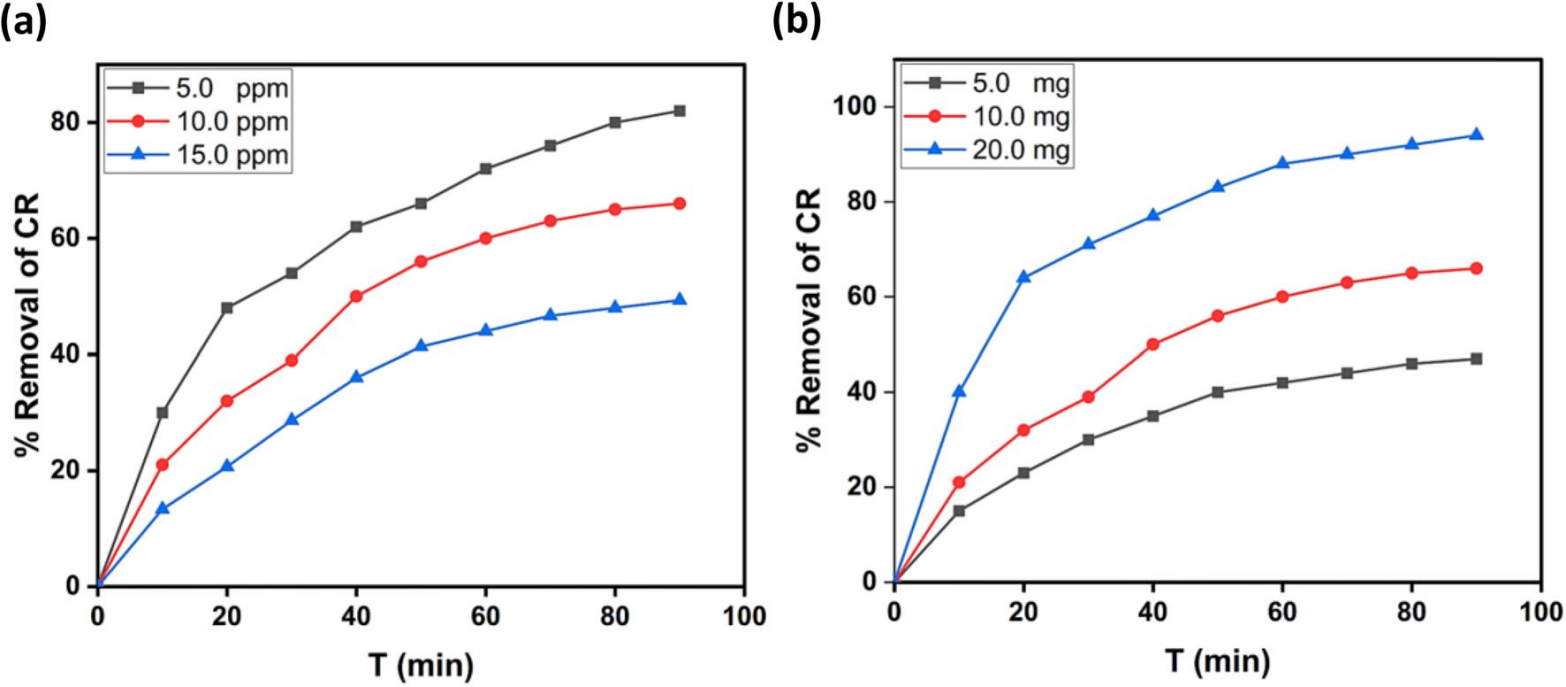
(**a**) Differences of removal percentage with contact time as a function at various initial CR concentrations (5, 10, and 15 mg/l) at pH 7 and 10.0 mg rGO-P nanocomposite. (**b**) Effect of the photocatalyst amount (in mg) on the removal efficiency of CR (50 ml CR solution, 10 mg/l at T = 25 °C and pH 7).

As the starting concentration of CR plays a crucial role associated with the removal process, the influence of CR's ionic strength was investigated by altering the CR's initial concentration while maintaining all other reaction conditions. Figure 6a illustrates the variance in removal percentage as a function of contact time for three distinct starting concentrations of CR (5, 10, and 15 mg/l).

The results show that the efficiency of CR degradation is inversely correlated with the CR's initial concentration, which can be effectively removed in the existence of the produced nanocomposite under UV light irradiation despite the starting concentrations being relatively high.

#### Effect of the rGO-P nanocomposite amount on degradation efficiency

As shown in Fig. 6b, the effect of a nanocomposite amount on the removal performance of CR under UV light was investigated by altering the quantity of the produced photocatalyst from 5, 10, and 15 mg against a fixed concentration of CR (10 mg/l). The findings demonstrated an improvement in removal efficiency when the photocatalyst amount was increased from 5 to 20 mg. The observed improvement in removal effectiveness with increased photocatalyst concentration might be due to an elevation in the active area or active sites of the photocatalyst to CR solution volume ratio^57^.

It’s worth to mention that our nanocomposite (rGO-P) shows a great potential as an efficient catalytic adsorbent in the removal of Congo red dye from waste water compared to other adsorbents reported in the literature, as it is obviously listed in Table 1.

**Table 1:** Comparison between rGO-P adsorbent with other adsorbents reported in the literature for the removal of Congo red (CR) dye at the same pH (i.e. pH = 3).

Adsorbent and/or Catalyst	Amount and/or concentration (mg/L)	Removal %	pH	Refs
Activated carbon and its regeneration	1000	~ 90	3	^58^
Bael shell carbon	50	92	3	^59^
MOP, CTAB-modified waste-orange peel-derived biosorbent	100	61	3	^60^
Azadirachta indica leaf powder	50	< 50	3	^61^
Activated carbon prepared from coir pith	4000	~ 65	3	^62^
rGO-P nanocomposite	200	78	3	current work

#### Kinetic studies

The following equation can be used to measure the rate of CR degradation:

1 $$- ln \, C_{t} /C_{0} = \, - \, kt$$

where: C_t_; the remaining concentration of CR, C_0_; the initial concentration of CR, *k*; the removal rate constant, and *t;* the removal time.

**Figure 7:**
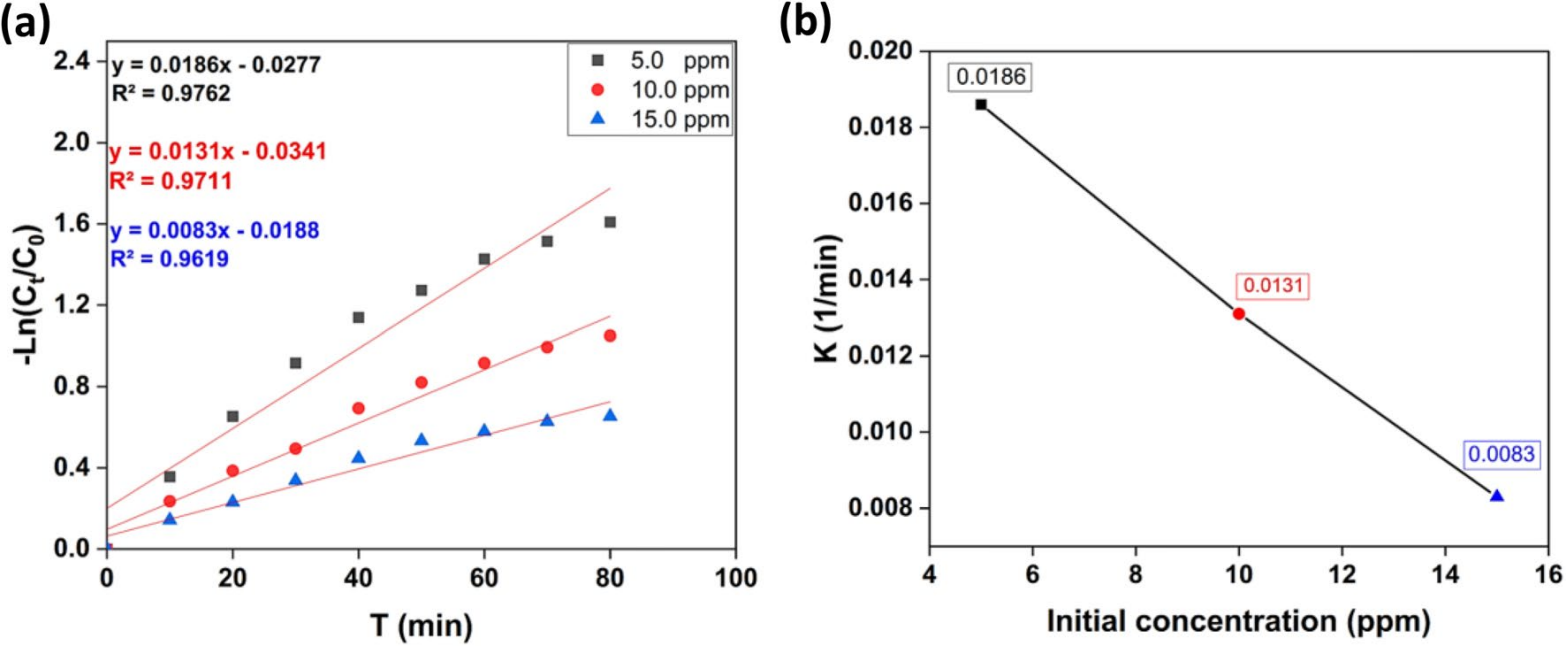
(**a**) Kinetics plots for linear fitting of data obtained from pseudo-first-order reaction model for Congo red degradation under UV light irradiation and 10 mg catalyst, 50 mL (of 5, 10, and 15 mg /L) dye concentration. (**b**) Relationship of apparent pseudo-first-order rate constants and initial concentration of CR.

Figure 7a depicts an association between −ln C_t_/C_0_ and *t*. The findings indicated that the kinetics of the removal process followed the laws for pseudo-first-order rate. Moreover, as revealed in Fig. 7b, an increase in CR concentration decreases the apparent pseudo-first-order rate constants. This dependence on CR concentration as a function of reaction rate constants is consistent with the literature^56,63^.

### Antimicrobial activity of the synthesized rGO-P nanocomposite

The disc agar distribution technique (as a screening approach) revealed that the synthesized rGO and rGO-P have a robust antibacterial effect against the examined microorganisms. The in vitro ZOI finding verified that the synthesized rGO-P nanocomposite exhibited its encouraged antibacterial activity against gram-positive *S. aureus and E. faecalis* as 22.1 and 21.8 mm ZOI, respectively, and against gram-negative *E. coli* and *S. sonnei* as 18.4 and 15.2 mm ZOI, respectively, as displayed in Table 2. It is worth considering that the antibacterial potency of rGO-P nanocomposite was significantly more powerful than porphyrin itself and bare-rGO, which suggested the synergistic potential between porphyrin and rGO. It is also worth noting that the rGO-P nanocomposite was less effective against gram-negative bacteria than gram-positive bacteria. Gram-negative cell walls are composed of layers of lipopolysaccharide, lipid, and peptidoglycan, while gram-positive cell walls are composed of very dense peptidoglycan forms^64^.

**Table 2:** Antimicrobial activity as the zone of inhibition ZOI (mm) of prepared samples against some pathogenic bacteria and yeast.

Test organism	Porphyrin	rGO	rGO-P composite	E/Nystatin
	ZOI (mm)	ZOI (mm)	ZOI (mm)	ZOI (mm)
*S. aureus (ATCC 25,923)*	9.^b^5 ± 0.14	14.^a^4 ± 0.23	22.^b^1 ± 0.24	22.5^a^ ± 0.29
*E. coli (ATCC 25,922)*	8.^c^6 ± 0.17	13.^b^4 ± 0.20	18.^c^4 ± 0.32	18.7^b^ ± 0.24
*Enterococcus faecalis (ATCC 29,212)*	9.^b^3 ± 0.24	12.^c^4 ± 0.17	^b^21.8 ± 0.72	6.5^d^ ± 0.29
*Shigella sonnei (ATCC 29,531)*	7.^d^5 ± 0.17	10.^e^5 ± 0.18	15.^d^2 ± 0.33	−ve
*Candida albicans (ATCC 14,053)*	12.^a^4 ± 0.20	11.^d^4 ± 0.14	24.^a^3 ± 0.17	12.6^c^ ± 0.18

*Values are means ± SD (n = 3). Data within the groups are analyzed utilizing one-way analysis of variance (ANOVA) accompanied by^a, b, c, d, e^ Duncan's multiple range test (DMRT).

*rGO = reduced graphene oxide and rGO-P = reduced graphene oxide loaded porphyrin, −ve = no ZOI had been measured, E = Erythromycin; 20 μg/ml antibacterial standard. Nystatin 10 μg/ml antifungal standard.

*LSD* Least significant differences.

### Mechanism of photocatalytic and antimicrobial activities the synthesized rGO-P nanocomposite

As reported previously^65,66^, the possible photocatalytic activity mechanism is described as follows. Changing the pH values affects photodegradation pathways such as explicit oxidation by positive holes in the valence band, hydroxyl radical attacks, and explicit decrease by electrons in the conduction band. It is expected that photocatalytic degradation will occur in the presence of rGO-P photocatalyst owing to the production of electron–hole pairs on the exterior of the employed photocatalyst owing to UV-irradiation. The reactive CR is oxidized by the holes' oxidative potential, which either combines with the OH groups to produce hydroxyl radicals or oxidizes the reactive CR to form a degradation product^67^. The following is a summary of the responses of CR and the used photocatalyst (Eqs. 2–5):

2 $${\text{rGO}} - {\text{P }} + {\text{ h}}\nu \to {\text{rGO}} - {\text{P NPs }}\left( {{\text{e}}^{ - }_{{{\text{CB}}}} + {\text{ h}}^{ + }_{{{\text{VB}}}} } \right)$$

3 $${\text{h}}^{ + }_{{{\text{VB}}}} + {\text{ rGO}} - {\text{P }} \to {\text{rGO-P NPs}}^{ + } \;{\text{(Oxidation of the compound}})$$

Or

4 $${\text{h}}^{ + }_{{{\text{VB}}}} + {\text{OH}}^{ - } \to {\cdot\text{OH}}$$

5 $${\cdot\text{OH + CR}}\;{\text{dye}} \to \left( {\text{Degradation products}} \right)$$

**Figure 8:**
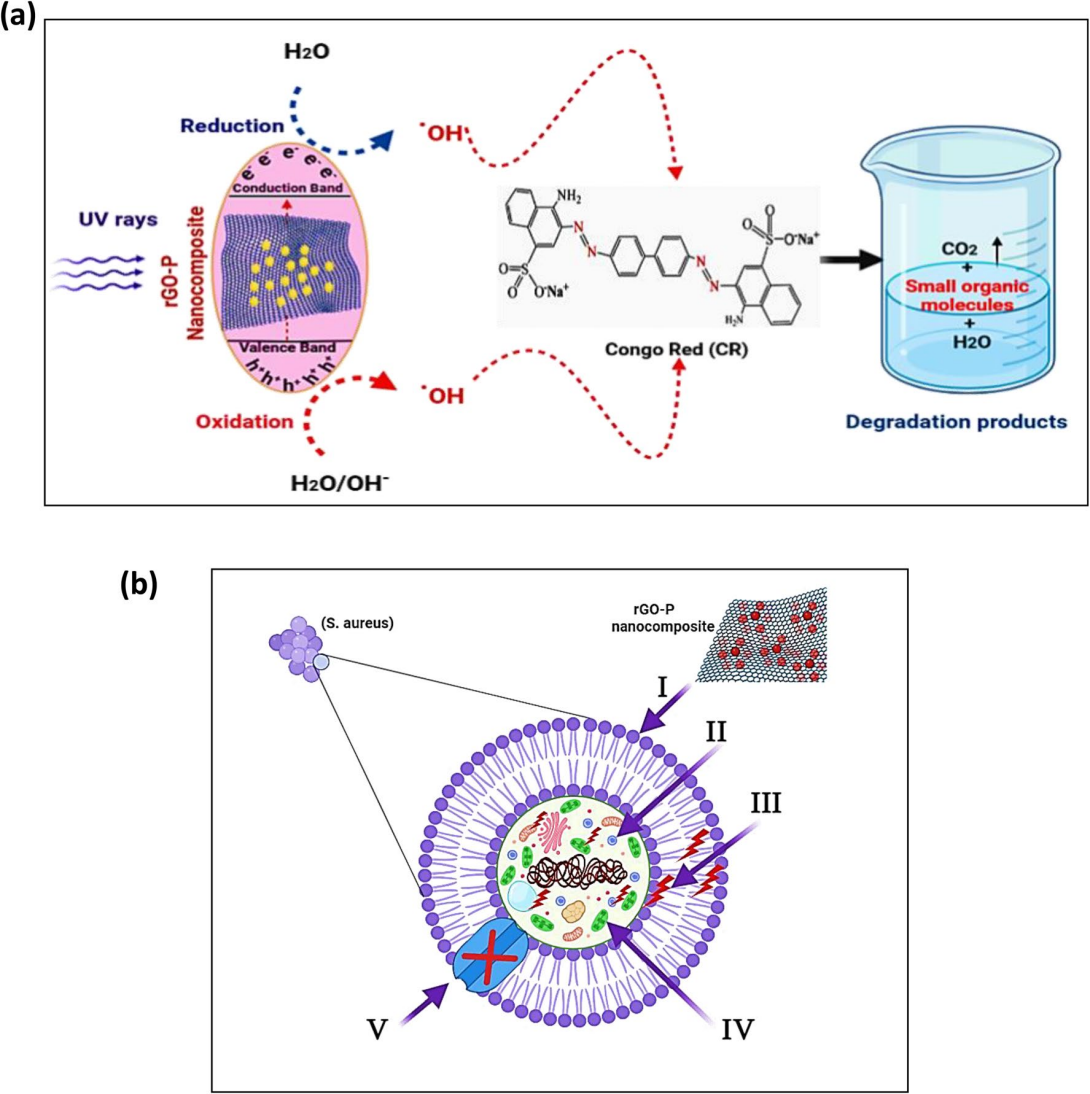
(**a**) Photocatalytic degradation mechanism of Congo red (CR) by rGO-P nanocomposite. (**b**) Schematic representation of the four main pathways underlying the antibacterial potential of the rGO-P nanocomposites: (I) the rGO-P nanocomposite adheres to and wrap the microbial cell surface, resulting in the release of porphyrin, causing membrane damage and altered transport activity. (II) rGO-P nanocomposite penetrates the microbial cells and interact with cellular organelles and biomolecules (such as plasmid DNA, ribosomes, chromosomal DNA, and mesosomes), affecting the respective cellular machinery. (III) rGO-P nanocomposite creates and increases ROS, leading to cell damage. (IV) rGO-P nanocomposite modulates the cellular signal system and causing cell death. (V) Finally, rGO-P nanocomposite blocks the ion transport from and to the microbial cells.

Figure 8a depicts the suggested interaction mechanism between the produced nanocomposite and CR. Charge carriers will be photogenerated, and redox processes will commence upon UV-light excitation of the rGO-P nanocomposite. The produced free radicals (such as ^**.**^OH and ^.^O^-^_2_) will then break down CR into tiny organic byproducts.

Moreover, graphene sheets decorated with porphyrins combine the outstanding properties of them and might result in some particular properties because of the synergetic effect between them. The practical applications of the photocatalyst degradation are limited due to the rapid recombination of photogenerated electrons and holes within photocatalysts. Considering the superior electron mobility and high specific surface area, rGO can be used as an efficient electron acceptor to enhance the photoinduced charge transfer and to inhibit the backward reaction by separating the evolution sites of hydrogen and oxygen^68^.

As reported previously^69^, during the photocatalytic reaction, the photo-induced electrons and holes reacted with oxygen (O_2_), water (H_2_O), and hydroxyl groups to generate reactive oxygen species (ROS) such as hydroxyl radicals (·OH) and superoxide radical anions (·O_2_^−^) with strong oxidation abilities. These ROS are the major responsible species for the degradation of persistent organic pollutants in wastewater.

Since there are currently no published data on the degradation of CR, it is necessary to do more research using gas chromatography-mass spectrometry (GC–MS) and high-performance liquid chromatography (HPLC) to examine the degradation products of CR in greater detail.

The schematic illustration in Fig. 8b shows the potential antimicrobial mechanism. Firstly, the rGO-P nanocomposites begin their activity by wrapping and adhering at the exterior surface of the microbial cells, causing membrane destruction and changing the transport potential. Then, the distribution of the porphyrin inside the microbial cell divides all intracellular structures (including plasmid, DNA, and other essential organelles). Afterward, cellular toxicity happens due to the oxidative stress created by the generation of ROS. Lastly, the nanocomposites block the ion transportation from and to the microbial cells.

## Conclusion

In this work, reduced graphene oxide doped with synthesized porphyrin (rGO-P nanocomposite) is conducted for their outstanding removal activity of Congo red (CR) dye and antimicrobial potential against gram-negative, gram-positive, and candida. SEM, HRTEM, Raman spectroscopy, XRD, mass spectrometry, UV–Visible spectroscopy, and ^1^H-NMR analysis techniques are used to characterize free-base porphyrin, rGO, and rGO-P nanocomposite. From the results, we suggested that the highest removal percent (i.e. 78%) of CR was achieved at pH 3.0. As a result, the net surface charge of the rGO-P nanocomposite is positive at this point, which attracts the negative charge of CR and promotes photocatalytic destruction of CR. The result of antimicrobial activity of all prepared samples reported that rGO-P nanocomposite has maximum activities against *S. aureus*, *Enterococcus faecalis*, and *C. albicans* with ZOI of 22.1, 21.8, and 24.3 mm, respectively. Finally, it was recommended that rGO-P nanocomposite is a promising antimicrobial agent against some pathogenic bacteria and yeast. Furthermore, it might be utilized as an ingredient in various cosmetics and medications for biomedical treatments. Due to their efficient photocatalytic activity, rGO-P nanocomposite may be used as a photocatalyst in environmental items such as water treatment from pollutant dyes and preserve our environment from dangerous contaminants.

## Supplementary Information

Supplementary Information.

## Data Availability

All data generated or analysed during this study are included in this published article [and its supplementary information files].
