# Supplementary material for: Synthesis and applicability of reduced graphene oxide/porphyrin nanocomposite as photocatalyst for waste water treatment and medical applications
Source: Sci Rep. 2022 Oct 12;12:17075. doi: 10.1038/s41598-022-21360-8 (PMC9556635; doi:10.1038/s41598-022-21360-8)
Supplement: Supplementary file 1 — Supplementary Information. [file 41598_2022_21360_MOESM1_ESM.docx]

**Supplementary Information**

**Synthesis and Applicability** **of Reduced Graphene Oxide/Porphyrin Nanocomposite as Photocatalyst for Waste Water Treatment and Medical Applications**

**Ahmed M. El-Khawaga ^a,b,*^, Hesham Tantawy ^c^, Mohamed A. Elsayed ^d^,**

**and Ahmed I. A. Abd El-Mageed ^e,f,*^**

*^a^Department of Basic Medical Sciences, Faculty of Medicine, Galala University, Galala City 43511, Suez, Egypt.*

*^b^Chemical Engineering Department, Military Technical College (MTC), Egyptian Armed Forces, Cairo, Egypt.*

*^c^Head of Chemical Engineering Department, Military Technical College (MTC), Egyptian Armed Forces, Cairo, Egypt.*

*^d^Head of School of Chemical engineering, Military Technical College (MTC), Egyptian Armed Forces, Cairo, Egypt.*

*^e^Chemistry Department, Faculty of Science, Galala University, Galala City 43511, Suez, Egypt.*

*^f^**Colloids & Advanced Materials Group, Chemistry Department, Faculty of Science, Minia University, Minia 61519, Egypt.*

**Corresponding Authors’ E-mails:*

*ahmed.elkhawaga@gu.edu.eg and* [*ahmed.abdelmageed@mu.edu.eg*](mailto:ahmed.abdelmageed@mu.edu.eg)

**Keywords:** *Reduced graphene oxide, Porphyrin, Antimicrobial activity, Photocatalysis, Nanocomposite.*

**Supplementary Table (s)**

**Table S1.** Chemicals used in the experimental work

| Chemicals | Purity | Source |
| --- | --- | --- |
| Graphite | 99.5% | NICE / India |
| Potassium Permanganate | 98% | Alpha Chemicals / India |
| Sulfuric Acid | 98% | Alpha Chemicals / India |
| Phosphoric Acid | 85% | Alpha Chemicals / India |
| Hydrochloric Acid | 36% | Alpha Chemicals / India |
| Hydrogen Peroxide | 35% | Alpha Chemicals / India |
| Ascorbic Acid | 98% | Alpha Chemicals / India |
| Ethanol | 99% | Alpha Chemicals / India |
| Congo red | ≤ 100% | Aldrich / Germany |

**Supplementary Figures**

**Figure S1.** Procedures for synthesizing 5,15-bisdodecylporphyrin (C12P) molecule.


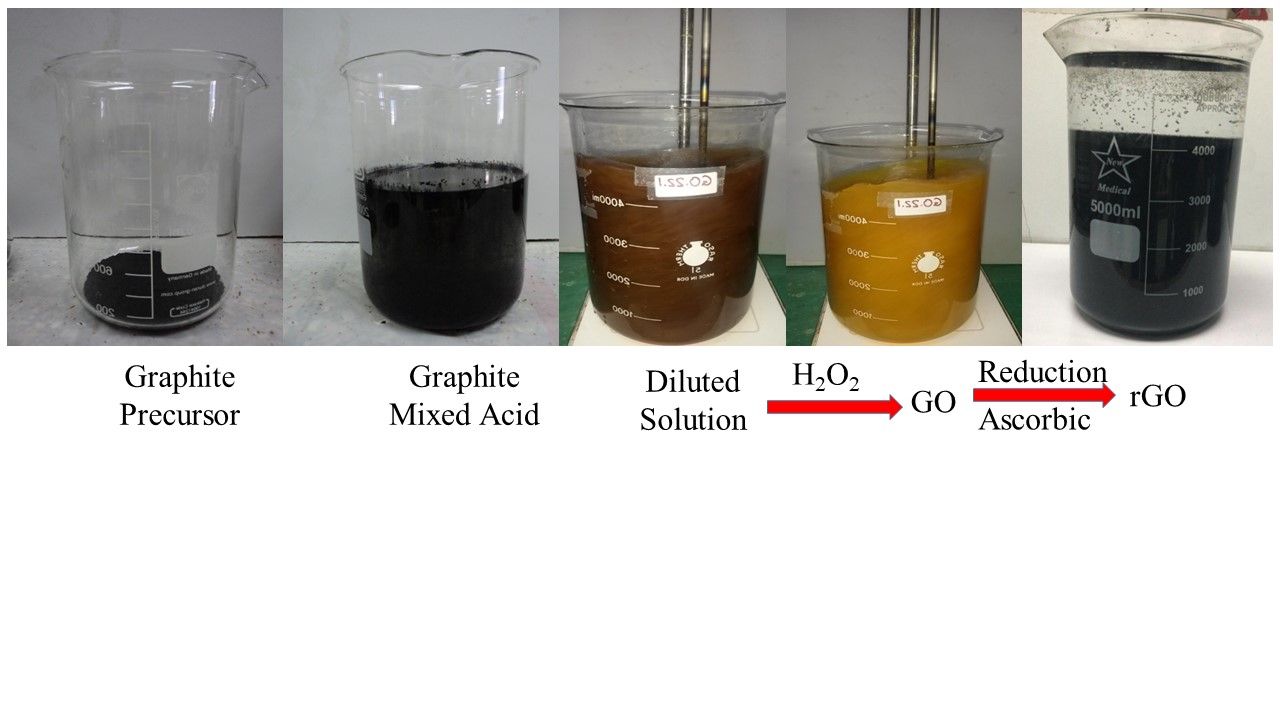


**Figure S2.** Synthesis of rGO.

**Figure S3.** Procedures for preparing rGO-P nanocomposite.

**Figure S4.** ^1^HNMR Spectrum of 5,15-bisdodecylporphyrin (C12P).

**Figure S5.** Mass Spectrum of 5,15-bisdodecylporphyrin (C12P).

**
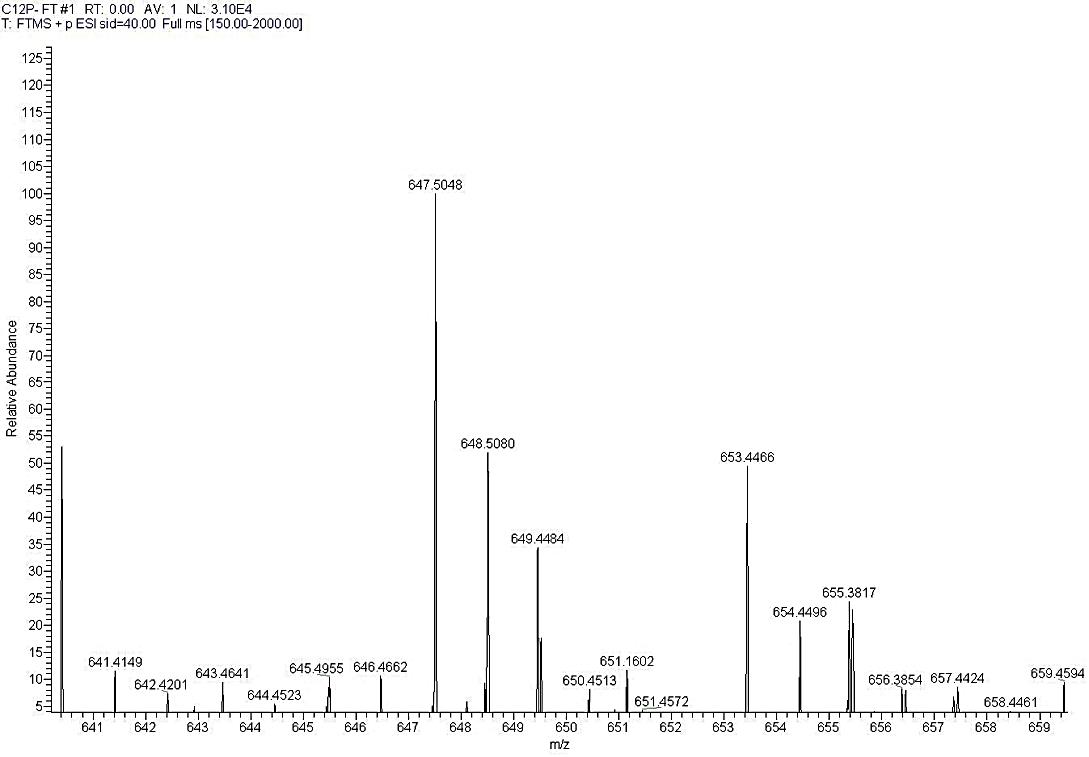
**

**Figure S6.** High resolution Mass Spectrum of 5,15-bisdodecylporphyrin (C12P).


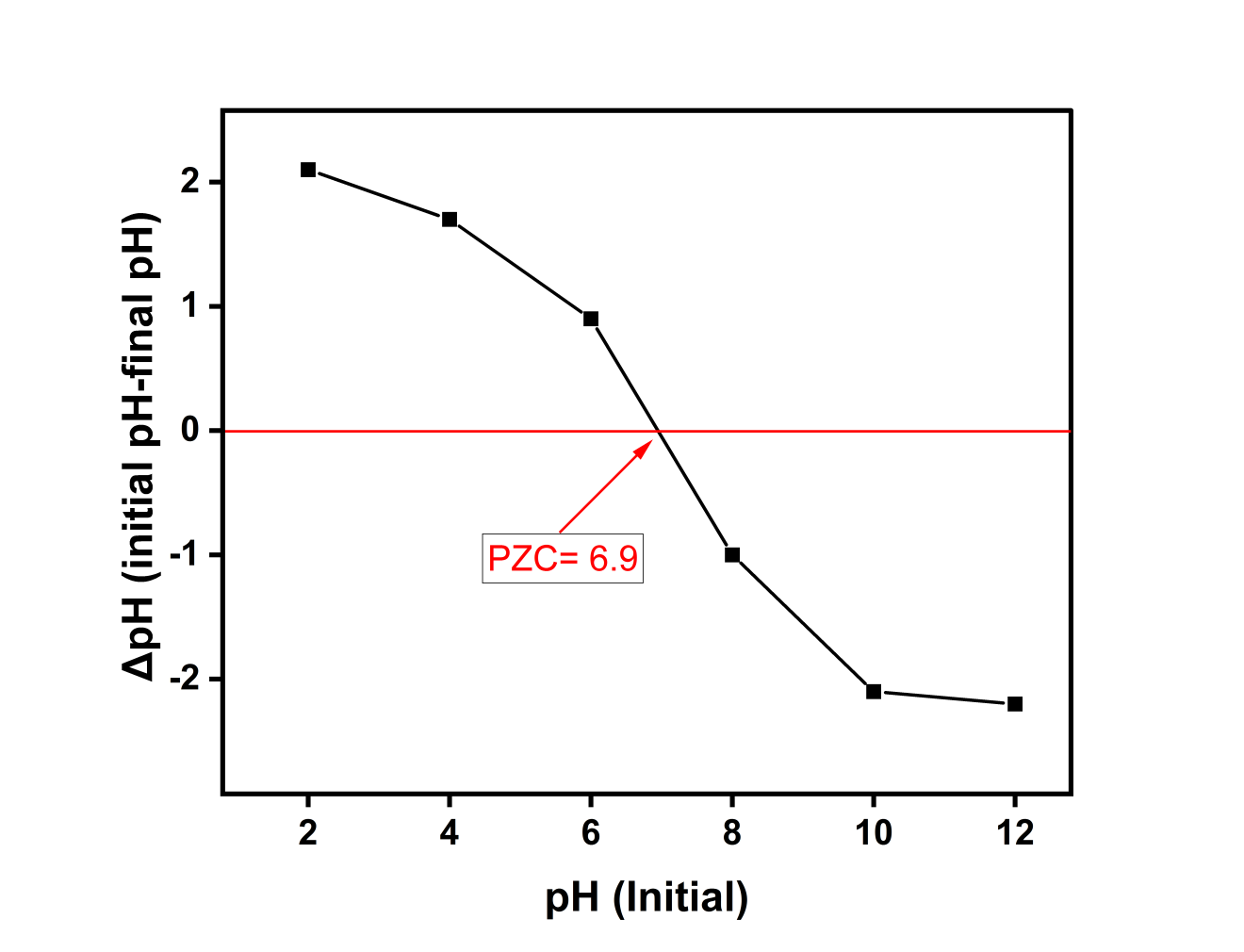


**Figure S7.** Point of zero charges (PZC) of rGO-P nanocomposite at different pH values.
